# Online Medical Teaching in China During the COVID-19 Pandemic: Tools, Modalities, and Challenges

**DOI:** 10.3389/fpubh.2021.797694

**Published:** 2021-12-20

**Authors:** Bin Su, Tong Zhang, Li Yan, Chaoyang Huang, Xiangpu Cheng, Chao Cai, Dan Cui

**Affiliations:** ^1^Beijing Key Laboratory for HIV/AIDS Research, Sino-French Joint Laboratory for Research on Humoral Immune Response to HIV Infection, Clinical and Research Center for Infectious Diseases, Beijing Youan Hospital, Capital Medical University, Beijing, China; ^2^Department of Education, Beijing Youan Hospital, Capital Medical University, Beijing, China

**Keywords:** online teaching, education modality, virtual classroom, medical education, COVID-19

## Abstract

**Background:** The novel coronavirus disease 2019 (COVID-19) pandemic has tested the ability of universities to provide a high-quality, safe educational experience for students due to campuses shutting down. As a result, online learning could shift from a traditional classroom teaching mode and make education accessible to students. Previous studies have used individual online teaching cases to exploit a variety of online learning tools to ensure the continuation of medical education during this difficult time in China. However, for the first time, we have conducted a systematic review of local online teaching approaches, existing challenges, and potential solutions.

**Purpose:** We present the issues and experience of conducting online medical teaching practices in China with the aim of communicating them to our peers in other countries or regions when examining the transition to e-learning during the COVID-19 pandemic and beyond.

**Methods:** We searched the keywords below from public databases and reviewed relevant publications reporting on medical online teaching in China during the COVID-19 pandemic to analyze and summarize the online tools, modalities, and challenges.

**Results:** We listed common online teaching tools and described a variety of online teaching modalities, as well as possible challenges. We also discussed potential solutions for those challenges, as well as the impact of the transition to online teaching on traditional education.

**Conclusion:** By investigating local online medical teaching in China, we present useful tools and modalities that have been successfully exploited in education during the difficult time of COVID-19, although some challenges remain. The exploration of the transition to online teaching or learning will likely continue to have a profound impact on traditional classroom teaching.

## Introduction

The outbreak of novel coronavirus disease 2019 (COVID-19) pneumonia evolved into a pandemic and has transformed a large part of daily life worldwide (1). The rapid spread of the disease has had a serious impact on school education, where face-to-face education has been damaged in many countries. According to a report by the United Nations, more than 191 countries and regions have closed their schools, and nearly 1.6 billion students have been affected by the pandemic (2). Many countries have taken steps to prevent infection through social distancing and distance education (3, 4). As one of the earliest responding countries, China also urged for campus closures in February 2020. To mitigate the impact of the pandemic on routine teaching activities, all levels of schools have proactively taken action to promote online education programs under the Chinese Ministry of Education initiative “Disrupted classes, undisrupted learning” (4–6).

Modern medical education encompasses a well-thought-out training system that covers highly structured curricula in a variety of preclinical and clinical environments (7). When considering the current state of medical education, traditional person-to-person educational didactics have now been challenged, like no other time before, given the current recommendations for public health measures to cancel on-site classroom teaching and limit regular clinical training activities. To heed the call for these recommendations, medical schools have switched to an online mode (8). By searching local public databases, namely the China National Knowledge Infrastructure (CNKI, https://www.cnki.net) and VIP Chinese Journal Platform (http://qikan.cqvip.com), we identified a few hundred publications about online teaching practices during the time of COVID-19. However, these publications are mostly based on individual cases exploring appropriate online teaching approaches with a variety of online tools. To systematically review and communicate the local online teaching situation in China, more specifically, we analyzed and summarized the implementation, challenges, and perspectives of online virtual classrooms for medical education programs during COVID-19 and beyond.

## Local Online Teaching Platforms and Applications

Since the beginning of the twenty-first century, information technology (IT) has been explored regarding its integration into the innovation of teaching approaches. For the past decade, online course resource platforms, such as Rain Classroom, have been established successively. Online teaching methods, such as micro-courses and flipped classrooms, have become useful supplements for offline teaching in schools (9–11). The speed of IT integration is accelerating, the range of disciplines involved is expanding, and the degree is deepening.

During the COVID-19 pandemic, online course platforms and applications have been further developed and implemented in distance teaching, including massive open online courses (MOOCs), Rain Classroom, WeChat, Moodle, QQ, and DingTalk (11). These virtual tools in China have been steadily developed and used as assistant teaching tools over the past decade. Asynchronously recorded broadcasting platforms, such as Rain Classroom, focus on home teaching, including videos, presentations, and exercises, which allow students to learn online and download educational materials (12). Instant messaging applications such as WeChat have been used for both sharing medical knowledge and collaboration (13, 14). Although these applications provide useful outlets for medical education, it is difficult to implement educational curricula due to a lack of organization. During COVID-19, a few innovative solutions have been developed to address these demands, such as Tencent Meeting and DingTalk, similar to the international video conferencing platform Zoom (15). In the face of the COVID-19 pandemic, millions of students and teachers have resorted to these applications or platforms to hold online classes (Table 1) (6). As revealed by a survey conducted at Tsinghua University on online learning platforms, Tencent Meeting has been the most commonly used (97%), followed by Rain Classroom (91%), and Zoom (86%) (17). However, this is not the same case for all other campuses, since there are no uniform standards for using online tools. Nevertheless, these platforms share common advantages in that they have been designed to be accessed either through PCs or smartphones. Students have therefore been able to learn with more convenience, without limitations of time or space (Table 2). Usually, these platforms have been selected to use together in online teaching, as they have different features or functions. For example, MOOCs and Superstar have been used to prepare prerecorded courses, and DingTalk and WeChat can be utilized to manage teaching disciplines and organize online discussions. In contrast, others such as Tencent Meeting and Rain Classroom can deliver online teaching through shared screens in real time or PowerPoint lectures. Their use in combination cannot only deliver learning content, but also keep teaching in good order.

**Table 1:** Major online learning platforms and applications.

**Online platform**	**Developer**	**Main uses**	**Accessibility**	**URL**
DingTalk (16)	Alibaba	Check-in/Online discussions/conferences	PCs/smartphones	https://www.dingtalk.com
Tencent Meeting (17)	Tencent	Conferences/Lectures	PCs/smartphones	https://meeting.tencent.com
WeChat (18)	Tencent	Online discussions	PCs/smartphones	https://weixin.qq.com
QQ (19)	Tencent	Online discussions	PCs/smartphones	https://im.qq.com/index
Superstar (20)	Century Superstar	Prerecorded courses	Smartphones	http://about.chaoxing.com
MOOC (21)	Open Source	Prerecorded courses	PCs/smartphones	http://www.umooc.com.cn
Rain classroom (9)	Tsinghua University	Online PowerPoint teaching	PCs/smartphones	https://pro.yuketang.cn
Moodle (22)	Open Source	Assignments/Discussions	PCs	https:/www.modlechina.org

**Table 2:** Advantages and disadvantages of online teaching.

**Advantages**	**Disadvantages**
•No time or geographic constraints •Cost-effective, available to distant learners •Student-centering teaching, able to promote student's autonomy •Less stress and beneficial for online discussions and asking questions •Students' learning progress can be automatically recorded	•Lack of face-to-face communication between teachers and students •Teachers and students are required to master computer skills •Need for PCs and internet access •Need for information technology (IT) support and necessary hosting resources •Online learning materials are usually updated quickly and have a higher rate of abandonment.

## Modalities of Virtual Teaching for Medical Education and Challenges

Within our community, academic programs have been stepping up to develop novel pedagogies for online learning. Dr. Song reported an online teaching modality for infectious disease education using MOOCs. The MOOCs provided a range of recorded courses for teaching about new and recurring infectious diseases. Along with problem-based learning (PBL), they highlighted the features of MOOCs in terms of feasibility, openness, and advancement to ensure that learners can master traditional theories and improve their clinical thinking ability (23). Li et al. described another online teaching method using prerecorded courses and a Superstar learning platform for infectious disease education. Meanwhile, they assigned a teaching secretary to communicate the class times and operations by asking and answering questions *via* WeChat and QQ applications. The trial, which lasted for one semester, showed that online learning can result in comparable achievements compared to traditional classroom teaching (24). Liu et al. explored the six-step BOPPPS teaching model when designing an online MOOC for infectious diseases. By bridging infectious disease cases, preset objectives, and the pre-assessment of students' ability, as well as participatory group discussions and post-class feedback, the model not only improved students' ability to analyze and solve problems related to the epidemic, but also deepened their sense of mission and active exploratory spirit (25). At Peking Union Medical College, teachers in each college chose Rain Classroom, Tencent Conference, and Zoom for online teaching, or used methods such as PowerPoint slides and voice messages to distribute course materials and kept in close contact with students through WeChat, Moodle, and other platforms, including pre-class preparation, assigning after-class homework, and hosting Q&A sessions to ensure the success of online teaching (26).

From these and other examples, online learning exhibits multiple benefits and advantages (Table 2). It breaks the limitations of time and space and can deliver large-scale teaching. Instructors and students can obtain plenty of online resources to assist in teaching and self-learning. Moreover, virtual teaching stresses problem-focused learning and eases group discussion, which is beneficial for students to develop self-learning and analytical ability (27, 28). However, the shift to online platforms poses a few challenges for medical education. The primary challenge is that most medical schools do not have sufficient time to prepare for online teaching on a large scale in a short period. Second, during transitions, network congestion, or crashes frequently occur due to a lack of adequate resources to handle sudden network loads. In addition, remote or rural areas do not tend to have stable network connections, and students in these areas may need hardware and IT support to participate in online classes. Third, as mentioned above, although E-learning platforms such as MOOCs provide a large amount of educational resources that come from schools themselves, peer institutions, or others published, it is difficult to probe and locate high-quality educational resources. For medical education resources, there are approximately 22 channels and websites providing more than 2,700 medical courses according to the list recommended by the National Center for Health Professions Education Development (NCHPED) (29). Some of them are presented in English, which creates learning barriers for instructors and students whose English language skills are not adequate. Fourth, a large proportion of instructors lack the confidence to execute online teaching, as they do not have such work experience (30). Other challenges include acquiring adequate clinical medical skills. On the basis of “best practices” reports from 40 medical schools in China, Jiang et al. presented 12 tips to help on-site medical classes move online under the pandemic, including teaching modes, infrastructure construction, platform integrations, and even educational policy decisions (30).

## Student Feedback on Online Teaching

To explore students' attitudes toward and perceptions of online teaching, a questionnaire survey was administered at Chongqing Medical University about the online teaching of infectious diseases through WeChat, QQ, and the SuperStar platform. The majority of students indicated their satisfaction with online teaching. Of 150 participants, over 90% expressed that online teaching can cultivate multiple abilities, such as self-learning, independent thinking, case analysis, and literature searching. Additionally, they could understand the knowledge of theory and clinical skills well (24). Similar results were obtained from other online teaching studies of different disciplines as well, such as medical imaging (20), obstetrics and gynecology (31), laboratory medicine (32), to name a few, indicating students could benefit from the student-centered online teaching and improve their multiple abilities mentioned above and even the spirit of teamwork. It's worthy to note that introduction of real-time chat tools extends students' questions beyond the classroom, no longer constrained by limited classroom time. Students can ask questions at any time when they find problems in their studies, and get detailed answers in a short time, and they can also view the questions asked by others. They can also help others solve problems, make up for the deficiencies in their own learning, and enhance self-learning depth (22).

## Thoughts on Medical Education in the Time of COVID-19 and the Post-Pandemic Era

Before the COVID-19 pandemic, distance online learning was not considered the major modality for education on campuses. Most online learning courses involve recorded lectures, which allow flexible attendance with the purpose of helping students understand the content taught in class. However, the occurrence of COVID-19 shifted pedagogy from traditional face-to-face classroom learning to online platforms, using synchronous or asynchronous live streaming techniques to deliver educational materials. Despite various challenges, such as internet streaming quality and coverage, online distance learning has been an efficient approach to learning about different educational topics. Some issues can be addressed with the widespread use of new technologies such as 5G telecommunication and virtual reality. Moreover, they can deliver high-quality, high-capacity transmission for live broadcasting, and interactions. Observations from online teaching in China show that students have revealed a more active attitude in learning when using vivid electronic materials such as images and videos in the teaching process (20). The COVID-19 pandemic aroused an interest in learning about virology. It has made virology more visible and provided content in real time to help students understand and contextualize aspects of emerging coronaviruses (33). In addition, by virtue of instant chat tools such as WeChat, students are more open to participating in group discussions due to the less intimidating environment (34, 35). Incorporating online Q&A sessions could also improve student engagement (36).

Regarding medical education, the main drawback is the impossibility of practicing and acquiring clinical skills. Telemedicine could provide another useful way for medical education to prepare students to participate in and develop competencies, including facilitating basic knowledge acquisition, improving decision-making, enhancing perceptual variation in anatomy lessons or three-dimensional simulations, improving skill coordination, practicing for rare or critical events, receiving team training, and strengthening psychomotor skills. Moreover, telemedicine can help medical students understand complex ethical, regulatory, and legal issues (37, 38). However, warranties should be provided based on learner feedback to address the potential lack of educator training in teaching telemedicine, such as communities of collaboration, professional credentialing standards, and automated guidance systems (37).

Mainstream views acknowledge the trend whereby online learning can replace traditional classroom learning, such as delivering theoretical knowledge except for clinical skills. It has multiple advantages, such as time saving, the flexibility of the classroom, and improved interactions with instructors and students (27, 39). However, counting on online learning for every aspect of medical education is impossible because on-site teaching is more dynamic and can avoid internet connectivity problems that probably occur in live broadcast teaching (40). In particular, online learning is no substitute for laboratory work training, which is usually accomplished person to person (41). Therefore, the blended approach of mixing traditional and e-learning classes would be the preferred way to deliver medical education in the future, and adopting future distance learning is significantly related to the degree of overall satisfaction (3, 42, 43). To maximize the benefits of both face-to-face and online teaching and to improve the efficacy of medical education, proven teaching modalities such as PBL and flipped classes are suggested (10, 44, 45). When setting up online learning courses, instructors can explore the topic to make students pursue self-paced enquiry under their own initiative. In addition, virtual teachers should conduct frequent assessments and check in by phone or other telecommunication formats with each student to enable them to catch up in courses (40).

Although there are differences in terms of cultural background and educational systems between different countries, higher education in all countries in the post-epidemic era is facing similar challenges and opportunities, such as the development of online or integrated teaching, teachers' professional development and information literacy improvements, the establishment of courses and training plans, and emergency management mechanisms. Like other disciplines, maintaining and strengthening medical education in the midst of and after COVID-19 will require thoughtful, concerted efforts on the part of governments, universities, and academic communities to collaboratively develop, implement, and fund long-term plans that elevate the voices of students and researchers in national policy decisions. In China, virtual teaching has significantly reshaped and innovated the teaching model and engagement with our medical trainees. In the future, with the advancement of technologies and the innovation of teaching modalities, we believe that medical training programs will benefit greatly from incorporating virtual learning platforms even beyond the time of COVID-19.

## Conclusions

By virtue of online learning platforms, students can access education anytime, anywhere. Online teaching has definitely played an important role in coping with course learning during the COVID-19 pandemic. Although isolation is a major disadvantage for online learning, we found through observation and analysis that this challenge can be overcome, and that online distance learning can be beneficial for both students and academic staff with the appropriate techniques and tools to support interactions and communication. To ensure the quality of online distance learning, China's 5G telecommunications network infrastructure can guarantee quick access to internet resources, as well as real-time communication. In addition, to minimize the requirement for computer skills and to ease online teaching management, efforts have also been made to preserve interactions in online distance learning courses. For example, in China, many schools have adopted popular local tools and platforms, such as using WeChat and MOOCs to perform online teaching management and deliver learning materials. Online teaching can be made more engaging and effective for both teachers and students through interactive teaching tools such as chat functions and videos. Of course, different regions and countries may utilize their own popular tools and platforms according to user habits or resource configurations, such as Zoom and Slack, to name a few widely used in Western countries for online teaching (46). In addition to hardware and software configuration, integration with appropriate teaching approaches is of great importance to online teaching. For example, flipped classroom teaching could be of great utility for encouraging independent learning and building student-centered teaching modalities. Just like exploring for more effective approaches through learning and absorbing advanced teaching modes and educational systems all over the world (47, 48), Chinese medical schools effectively integrated local resources with foreign teaching modes for dealing with the challenge of the COVID-19 pandemic imposed to medical education. In the future, further surveys are required to be conducted to monitor the development of online learning as this pandemic carries on, including the improvements in shortcomings of the current online distance learning for medical students. We also hope to continuously share these valuable educational experience with peers in the medical community.

## Author Contributions

BS conceptualized the study. BS, LY, CH, and XC searched the literature, selected studies, and extracted the data. BS, TZ, CC, and DC contributed to the analysis and interpretation of the data and provided important scientific input. BS analyzed the findings and wrote the manuscript. BS, CC, and DC supervised the whole study. All authors collaboratively discussed key decisions throughout the course of the review, provided critical feedback on preliminary manuscript, and approved the final version.

## Funding

This work was supported by the National Natural Science Foundation of China (NSFC, 81974303 and 81772165 to BS), the Scientific Research and Cultivation Program of Beijing Municipal Administration of Hospitals (PG2019029 to DC), the Climbing the peak (Dengfeng) Talent Training Program of Beijing Hospitals Authority (DFL20191701 to TZ), the China Primary Health Care Foundation-Youan Medical Development Fund (BJYAYY-2020PY-01 to BS), the Research Project of Education and Teaching Reform of Capital Medical University (2020JYJX180 to XC), the Online Open Course Construction of Capital Medical University (Infectious Diseases) (WL_78210157 to XC), and the Beijing Key Laboratory for HIV/AIDS Research (BZ0089). The funders had no role in study design, data collection and analysis, decision to publish, or preparation of the manuscript.

## Conflict of Interest

The authors declare that the research was conducted in the absence of any commercial or financial relationships that could be construed as a potential conflict of interest.

## Publisher's Note

All claims expressed in this article are solely those of the authors and do not necessarily represent those of their affiliated organizations, or those of the publisher, the editors and the reviewers. Any product that may be evaluated in this article, or claim that may be made by its manufacturer, is not guaranteed or endorsed by the publisher.
